# Effect of the pandemic on prehospital management of patients with mental and behavioral disorders: a retrospective cohort study

**DOI:** 10.3389/fpubh.2023.1174693

**Published:** 2023-09-13

**Authors:** Nikolaos Kintrilis, Natasza Blek, Sergiusz Blek, Aleksandra Olkiewicz, Jerzy Robert Ladny, Lukasz Szarpak

**Affiliations:** ^1^Infectious Disease Unit, General Military Hospital of Athens, Athens, Greece; ^2^Institute of Outcomes Research, Maria Sklodowska-Curie Medical Academy in Warsaw, Warsaw, Poland; ^3^Department of Neurology, Wolski Hospital, Warsaw, Poland; ^4^Research Unit, Polish Society of Disaster Medicine, Warsaw, Poland; ^5^Department of Emergency Medicine, Bialystok Medical University, Białystok, Poland; ^6^Henry J.N. Taub Department of Emergency Medicine, Baylor College of Medicine Houston, Houston, TX, United States

**Keywords:** mental, behavioral, prehospital management, emergency medical service, COVID-19, pandemic

## Abstract

The novel severe acute respiratory syndrome coronavirus (SARS-CoV-2) infection and the accompanying coronavirus disease (Covid-19) have shifted the priority of human and technical resources toward their handling, thus affecting the usual standards of care for populations diagnosed with other clinical entities. The phenomenon becomes even more apparent in patients with presenting symptoms of mental and behavioral disorders, a category already vulnerable and underrepresented in regard to its prehospital approach and management. For the purposes of the current retrospective cohort study, we used records of the Polish National Emergency Medical Service Command Support System for the time period between April 1, 2019 and April 30, 2021, the official register of medical interventions delivered in Poland by Emergency Medical Services (EMS). We aimed to examine the potential impact of the COVID-19 pandemic across the Masovian Voivodeship on individuals seeking medical care for mental and behavioral disorders pertaining in the “F” category of the International Statistical Classification of Diseases and Related Health Problems, 10th Revision (ICD-10). We examined the individuals’ baseline characteristics, prehospital vital parameters and EMS processing times in a population of 59,651 adult patients (04/2019–03/2020, 28,089 patients, 04/2020–03/2021, 31,562 patients) handled by EMS teams. Compared to pre-COVID-19, EMS personnel handled fewer patients, but more patients required mental and behavioral care. Throughout the duration of the pandemic, all prehospital time periods were significantly delayed due to the increased time needed to prepare crew, vehicles, and technical equipment to ensure COVID-19 prevention and overcrowding in Emergency Departments (EDs).

## 1. Introduction

The International Statistical Classification of Diseases and Related Health Problems (ICD) is a taxonomy of disease used worldwide for health management and clinical purposes, serving as a global communication tool among healthcare providers all over the world. Published by the World Health Organization, it includes a huge plethora of diseases, symptoms, and clinical complaints and is used daily not only for statistical and epidemiology purposes, but also to aid clinical decision-making and the management of patients all over the world. Within the ICD, codes starting with the letter F (F01-F99) encompass the spectrum of mental, behavioral, and neurodevelopmental disorders (1). Mental health or psychiatric emergencies are defined as acute incidents stemming from disturbances in thought, mood, or behavior that can present an imminent danger for the affected person or their environment if left without attention (2, 3).

Although data on the epidemiology of ED attendances for mental health-related issues are limited, a systematic review and meta-analysis of observational studies conducted in seven countries revealed that the number of ED attendances by patients with common mental health conditions is not insignificant, accounting for 4% of the total patient population. Even more concerning is the fact that among this group, one third of individuals sought help because of self-harm or suicidal ideation, and more than half of the patients were eventually admitted (4). Another cross-sectional analysis from England, the first national study regarding mental health attendance, presented similar findings, attributing 4.2% of ED attendances to mental health problems, a third of which were among patients who were eventually admitted to the hospital. Interestingly enough, two thirds of the individuals seeking help needed ambulance transportation (5). Mental health is a burning topic among the Polish population, with more and more children and adolescents requiring treatment for anxiety and depression in the country, and rates for both major depression and simple mood disorders more than doubling over the last 15 years (6). Furthermore, the pandemic yielded negative results when it came to the mental health, positive thinking, and sense of well-being of the Polish inhabitants, with feelings of loneliness, anxiety, and depression being reported at greater rates compared to the pre-pandemic era (7, 8).

Data regarding waiting times for psychiatric disorder patients are inconclusive, with some studies reporting swift management of mental health problems compared to physical illness (9, 10), while others mention a longer length of stay in the ED and a more complicated procedure in order to be admitted and offered the required therapeutic measures (11). Since poor mental health seems to correlate with increased ED attendance (12), prompt management of psychiatric conditions appears of utmost importance in the setting of efficient healthcare personnel and material utilization.

The ongoing pandemic of COVID-19 has invaded all factors of public and social life during the last 3 years, with profound effects on how healthcare for other medical entities is delivered. Not only do ED physicians have to face an increased workload due to the additional coronavirus cases presenting for examination and treatment, but also effectively organizing EDs in order to protect working staff, patients, and other visitors from the spread of the virus has been a challenge for hospitals worldwide (13, 14). Poland, a wide country with a multitude of middle-range populated towns and their respective county hospitals (15) has not remained unaffected by the above situation, with the country’s nationals recognizing a decline in the offered healthcare services compared to before the pandemic era (16). With the Ministry of Health officially deciding to allocate healthcare provisions toward the pandemic, transforming medical facilities into infectious disease units, for example (17), this came as no surprise.

Emergency Medical Services (EMS) teams represent the starting point of care for all patients requiring transport to an ED, whether they present with physical or behavioral complaints, and their role is vital in the stabilization and possible first therapeutic measures for each individual’s condition. Upon arrival at the scene, the rescue team engages in triage along with vital sign examination (blood pressure, respiration rate, pulse and rhythm, and measurement of blood glucose values), followed by stabilization and if needed, basic life support procedures until arrival at an organized medical setting. The primary aim of this study was to examine the diagnostic and therapeutic practices utilized for patients seeking medical treatment for mental and behavioral disorders falling under the “F” category of the International Statistical Classification of Diseases and Related Health Problems, 10th Revision (ICD-10), specifically focusing on the prehospital stage and the general characteristics of these patients. However, due to the occurrence of the COVID-19 pandemic during the course of the study, the objective was modified to include a comparative analysis of pre- and post-pandemic cohorts. Consequently, two distinct time periods were investigated. This study was conducted with the purpose of evaluating the impact of the COVID-19 epidemic on individuals diagnosed with mental, behavioral, and neurodevelopmental disorders, specifically examining their baseline characteristics, prehospital vital parameters, clinical status, and emergency medical services (EMS) processing times within the Masovian Voivodeship.

## 2. Materials and methods

### 2.1. Study design

Data for the purposes of the present study originated from the Masovian Voivodeship, which comprise the biggest voivodeship of Poland in terms of both geographical territory and inhabitants, including the country’s capital and largest city, Warsaw. With a geographical territory of 35,579 km^2^ and almost 5.5 million residents (18), the province utilizes its emergency healthcare services for all types of emergent health-related issues. Of note, an analysis of emergency attendances within the province revealed that the most common health issues for which inhabitants sought help were cardiovascular diseases. However, the percentage of people seeking help for mental health-related health issues was also high compared to the ones mentioned in the introduction, rising to more than 10% (19).

A retrospective cohort analysis of adult patients seeking emergency health service help was conducted, based on the ICD-10 categorization of their presenting symptomatology. Analysis included patients with at least one presenting symptom pertaining to the “F” codes of the ICD-10 taxonomy coded by EMS crew, with the total number of patients receiving prehospital care for such complaints for the period between April 1, 2019 and March 31, 2021 being 59,651. For the purposes of comparison between the pre-pandemic and pandemic eras, the studied period was further divided into two sub-periods, specifically 01/04/2019 to 31/03/2020 (pre-COVID-19 period) and 01/04/2020 to 31/03/2021 (COVID-19 period). Elimination criteria included being under the age of 18 and not having any information needed for the current study’s analysis. The study adhered to the World Medical Association Helsinki Declaration regarding ethical principles for medical research involving human subjects (20) and strengthening the reporting of observational studies in epidemiology (STROBE) guidelines for observational studies (21). The research was approved by the Institutional Review Board of the Polish Society of Disaster Medicine (approval number 01.12.2022.IRB).

### 2.2. Data collection

The Polish National Medical Service Command Support System, an electronic register of emergency health services dispatch and medical interventions performed by EMS teams, was used for data collection. Within the records of the aforementioned time periods, we identified all cases bearing at least one initial diagnosis pertaining to the F00–F99 taxonomy according to the International Statistical Classification of Diseases and Related Health Problems revision 10 (1). The analysis was restricted to the prehospital management of the individuals since subjects were redirected to a plethora of different hospital EDs. For each individual, gender, age, time needed to approach, vital parameters, initial medical diagnoses, medical actions performed, and result of the intervention were parameters pulled from the database and subjected to statistical analysis.

### 2.3. Statistical analysis

Statistical analyses were performed using the Statistical Package for Social Science (SPSS) data suite version 26.0 (SPSS, IBM, Armonk, United States). Arithmetic means, extreme values, and standard deviations were calculated for quantitative variables, as were frequencies of occurrence for quantitative values. Qualitative variables were compared between groups using the chi-square (χ^2^) test or, for small samples, the Fisher exact method. For the comparison of means, Welch’s *t*-test was used. A statistical significance level of *p* = 0.05 was chosen for this study.

## 3. Results

### 3.1. Patient demographics and baseline characteristics

From April 2019 to March 2020, medical emergency teams from the Mazovian Voivodeship performed 435,562 patient encounters, of which 28,089 interventions were carried out on patients with psychiatric diagnoses—6.45% of all EMS interventions. From April 2020 to March 2021, EMS teams from the Mazovian Voivodeship performed 386,764 patient encounters, of which 31,562 were carried out on patients with psychiatric diagnoses (Table 1), which constituted 8.16% of all EMS interventions in the pandemic period. Statistically significant increases in prevalence were noted in the categories of Addictions F10-F19, Psychotic disorders F20-F29, Neurotic disorders F40-F48, and Other disorders of children and adolescents F90-F98.

**Table 1:** ICD-10 F codes: mental, behavioral, and neurodevelopmental disorders—diagnoses used by emergency medical services teams.

	Pandemic (*n* = 31,562)		Pre-pandemic (*n* = 28,089)		*N*	*p*	Test
	*n*	%	*n*	%			
Organic disorders F00–F09	3,600	11.4%	3,217	11.5%	6,817	0.86	Chi2
Addictions F10–F19	8,534	27.0%	7,946	28.3%	16,480	**<0.001**	Chi2
Psychotic disorders F20–F29	3,258	10.3%	2,696	9.6%	5,954	**<0.01**	Chi2
Affective disorders F30–F39	2,152	6.8%	1,920	6.8%	4,072	0.93	Chi2
Neurotic disorders F40–F48	7,383	23.4%	6,350	22.6%	13,733	**0.023**	Chi2
Eating disorders F50–F59	110	0.3%	90	0.3%	200	0.55	Chi2
Personality disorders F60–F69	413	1.3%	348	1.2%	761	0.45	Chi2
Intellectual disability F70–F79	375	1.2%	429	1.5%	804	**<0.001**	Chi2
Disorders of psychological development F80–F89	246	0.8%	186	0.7%	432	0.092	Chi2
Other disorders of children and adolescents F90–F98	1,655	5.2%	1,371	4.9%	3,026	**0.044**	Chi2
Unspecified mental disorder F99	4,884	15.5%	4,392	15.6%	9,276	0.59	Chi2

Statistical significance.

As a result, the covered period for the needs of the present study came up with a total of 59,651 patients who notified EMS teams with complaints of a mental or behavioral disorder. When divided into two periods, pre- and during the pandemic period, *n*1 = 31,562 patients sought help during the time interval of 01/04/2020 to 30/04/2021, and *n*2 = 28,089 patients sought help during the time interval of 01/04/2019 to 31/03/2020. The mean patient age across the two sub-periods did not differ significantly, namely 45.7 ± 20.4 and 45.4 ± 21.4 years, respectively. During Pandemic, here was an increase in interventions occurring more frequently at home. Secondly, the police were notified more often during EMS interventions in the time of Pandemic. There was a noticeable change in the type of transportation used by medical emergency teams (EMS) during the pandemic—the majority of EMS interventions involved basic ambulances. Moreover, it was observed that a larger proportion of patients were being transported and admitted to hospitals before the pandemic. Full patient demographics and baseline characteristics can be found in Table 2.

**Table 2:** Baseline characteristics.

		Pandemic (*n* = 31,562)		Pre-pandemic (*n* = 28,089)		*N*	*p*	Test
Age, mean		45.7 (±20.4)		45.4 (±20.4)		56,404	0.087	Welch
		*n*	%	*n*	%			
Sex	Male	14,857	54.95%	13,335	56.43%	28,192	**<0.001**	Chi2
	Female	12,180	45.05%	10,296	43.57%	22,476		
Scene	Home	24,441	77,45%	20,477	72,90%	44,918	**<0.001**	Chi2
	Public	6,145	19,47%	6,306	22,45%	12,451		
	Traffic	331	1,05%	418	1,49%	749		
	Work	427	1,35%	453	1,61%	880		
	School	170	0,54%	398	1,42%	568		
	Farming	43	0,14%	37	0,13%	80		
Police was notified	No	21,943	69.52%	20,569	73.23%	42,512	**<0.001**	Chi2
	Yes	9,619	30.48%	7,520	26.77%	17,139		
Fire department was notified	No	30,732	97.37%	27,370	97.44%	58,102	0.59	Chi2
	Yes	830	2.63%	719	2.56%	1,549		
MRT type	B	25,888	82.02%	22,259	79.24%	48,147	**<0.001**	Chi2
	S	5,674	17.98%	5,830	20.76%	11,504		
Patient admission to the ED	Admission	20,078	63.61%	19,022	67.72%	39,100	**<0.001**	Chi2
	Refusal	85	0.27%	102	0.36%	187		
	Not transported	11,399	36.12%	8,965	31.92%	20,364		

Statistical significance.

For the time period studied, vital parameters were as follows (Table 3).

**Table 3:** Vital parameters.

		Pandemic (*n* = 31,562)	Pre-pandemic (*n* = 28,089)	*n*	*p*	Test
Systolic blood pressure in mmHg, mean		138 (±20.4)	137 (±20.6)	50,200	**<0.001**	Welch
Diastolic blood pressure in mmHg, mean		82.4 (±11.5)	82.2 (±11.2)	50,133	0.45	Mann–Whitney
Heart rate (/min), mean		92.7 (±16.8)	92.9 (±17.3)	54,434	0.13	Welch
Respiratory rate, mean		16.1 (±4.58)	16.3 (±4.75)	56,212	**<0.001**	Mann–Whitney
Blood oxygen saturation, mean		97.2 (±4.47)	97.2 (±4.52)	54,008	**<0.001**	Mann–Whitney
Blood glucose in mg/dL, mean		130 (±47.4)	129 (±48.0)	23,867	**<0.001**	Mann–Whitney
GCS—sum	15	26,355 (85%)	23,354 (85.3%)	49,709	0.28	Chi2
	9–14	4,468 (14.4%)	3,850 (14.1%)	8,318		
	3–8	178 (0.6%)	176 (0.6%)	354		

Statistical significance.

### 3.2. Prehospital time intervals

As seen in Table 4, all prehospital time intervals were considerably delayed during the pandemic period. More specifically, the median time from call to arrival of the emergency team before the pandemic was 13.5 min, rising to 15.9 min after the pandemic, a statistically significant difference (*p* < 0.001). Total time of medical team intervention until patient admission to the ED also differed significantly before and during the pandemic, with total times rising from 50.2 to 60.9 min (*p* < 0.001).

**Table 4:** Prehospital time intervals.

	Pandemic (*n* = 31,562)	Pre-pandemic (*n* = 28,089)	*n*	*p*	Test
Time from call to ED admission, median [Q25–75]	60.9 [43.9; 88.6]	50.2 [37.1; 69.2]	45,391	**<0.001**	Mann–Whitney
Time from call to contact, median [Q25–75]	15.9 [10.9; 23.2]	13.5 [9.35; 19.2]	59,651	**<0.001**	Mann–Whitney
Time from contact to ED admission [Q25–75]	42.4 [28.8; 64.6]	35.0 [24.0; 51.4]	45,384	**<0.001**	Mann–Whitney

Statistical significance.

## 4. Discussion

The present study regards changes in the prehospital management of adult patients with mental and behavioral disorders by EMS teams in the largest voivodeship of the Polish territory before and during the ongoing COVID-19 pandemic. To begin with, the overall number of individuals reaching out for emergency care due to mental and behavioral problems rose by an absolute number of 3,473 people, or 12.4% during the pandemic as compared to before. Since the total number of EMS team encounters dropped during the pandemic era, the increase in the percentage of mental health emergencies is even more profound (6.45% of all EMS encounters were mental health-related before, whereas the percentage rose to 8.16% during the pandemic).

Early studies from the lockdown era in the United Kingdom and other European countries showcased an increase in psychological distress among the population, with women and adolescents being the most commonly affected groups (22–25). Other predisposing factors beyond age and gender included unemployment and financial instability, as well as poor social support. Pre-existing mental health issues, specific personality traits, and maladaptive personality were also listed as risk factors (22). At the same time, the deterioration of mental health levels was extremely profound among the younger generations, with children, teenagers, and adolescents presenting increased levels of feelings of loneliness and mental malaise (26, 27). On the other hand, no substantial mental health changes were observed among the older adults within the European region, with older people being more concerned about the infection itself than isolation and loneliness feelings (28).

A large systematic review of studies from 63 countries found a pattern in the use of mental health emergency services that corresponded to the different pandemic phases. More specifically, initial ED and secondary healthcare center attendances appeared limited, probably owing to the initial lockdown phase and general public hesitancy about healthcare service utilization during the first pandemic phase. Later on, attendance increased as feelings of loneliness and psychological distress increased among the population (29, 30). Of note, though, an Italian study focusing on the types of psychiatric emergencies during the lockdown phase showed an alteration in the clinical profile of patients seeking emergency help for mental health reasons, with non-severe mental issues dominating over severe ones (31).

The most important finding from our analysis regards prehospital management times, which appear to have significantly increased although the EMS workload in terms of absolute numbers of patients was reduced during the pandemic period. More specifically, not only did the median time from call to arrival of the emergency team rise to 15.9 [10.9, 23.2] min after the pandemic compared to 13.5 [9.35, 19.2] min before, but also a statistically significant difference was also observed in the total time of medical team intervention until handling the patient at the ED (60.9 [43.9, 88.6] vs. 50.2 [37.1, 69.2] min), meaning that each dispatch took more time. Literature suggests increases in prehospital management and arrival on-scene times among healthcare systems around the globe (32–36), with multiple reasons being suggested to explain the phenomenon of reduced calls but increased workload.

First and foremost, emergency crew response times appear to have increased during the pandemic due to the increased time needed to prepare crew, vehicles, and technical equipment before shifts and between calls, as well as the time needed to distribute patients in ED rooms, with most hospitals dividing patients into febrile and afebrile, or high- and low-suspicion COVID-19 patients. Disinfection, preparation, and generally organizing each delivery take more time per case than before, resulting in increases in total handling times (37).

Our additional observations pertain to specific aspects associated with the nature of the interventions. With people spending more time indoors due to lockdowns and social distancing measures, it is understandable that home-related incidents would see a rise.

Increase in police interventions could be attributed to several factors, such as increased tensions within households, strained mental health conditions, or heightened concerns for personal safety during uncertain times.

There was a noticeable change in the type of transportation used by medical emergency teams (EMS) during the pandemic—the majority of EMS interventions involved basic ambulances. The shift regarding ambulance type might be attributed to the need for streamlined and efficient healthcare response during the pandemic, as well as the focus on providing immediate medical attention rather than specialized services.

The fact that less proportion of patients were transported to the hospital could potentially be linked to the fear of infection and the limited availability of hospital resources during the pandemic. This factor may also have influenced the overall reduction in the number of interventions during the pandemic, as did the widespread introduction of telemedicine consultation options for general practitioners. Another possible explanation for the decrease in patients being transported to hospitals during the pandemic could be attributed to the improved condition of patients after receiving medical attention from the ambulance teams. Upon arrival, the EMS teams may have administered treatments like anti-anxiety medication or provided other necessary interventions, leading to a clinical improvement in the patients’ conditions. As a result, some patients may have experienced relief from their symptoms and opted not to seek further hospitalization.

Additionally, EMS teams as well as ED physicians have assumed new and varied healthcare roles during the pandemic, owing to the overall increased healthcare needs. Provision of vaccinations, managing COVID-19 admissions, and caring for post-COVID-19 sequelae belong to the new spectrum of conditions being managed by most specialties, especially internists, pneumologists, and intensive care unit personnel, all specialties commonly involved in the emergency rooms (38, 39). It is essential to note that the COVID-19 pandemic has had a significant impact on the mental health of EMTs, resulting in elevated levels of stress and anxiety (40), which have led to decreased self-efficacy and sleep quality (41).

What remains unquestionable, though, is that the management of non-COVID-19 emergencies during the pandemic era has changed for the worse, with studies showing an important disruption not only in referral of patients to the ED rooms, but also to the overall mortality of non-infectious emergencies (42, 43). It is obvious that a structured approach to the redesign of prehospital and ED management of both infectious and non-infectious patients is critical until the current pandemic subsides and healthcare systems resume their normal rhythm.

### 4.1. Limitations

This investigation is subject to several limitations that warrant consideration. Firstly, our study population consisted of individuals who received prehospital diagnoses, without subsequent confirmation or follow-up regarding the presence of suspected disorders. Moreover, it is important to note that there may be missing data pertaining to certain patients, as our analysis was based solely on the information provided by EMS providers. This limitation significantly hampers the interpretation of results, as it fails to capture the frequent utilization of outpatient services by individuals experiencing mental health difficulties. There was a moderate level of missing data in our patient demographics and vital signs.

## 5. Conclusion

We conducted a retrospective cohort analysis of the Polish national registry of medical interventions performed by EMS teams in the Mazovian province in order to assess possible changes in prehospital care delivery between the pre-pandemic and the pandemic era. More specifically, we screened the National Emergency Medical Service Command Support System for mental and behavioral health complaints by recruiting all ICD-10 diagnoses belonging to this spectrum (diagnoses with ICD-10 codes starting with “F”). Compared to the pre-COVID-19 period, the overall number of patients handled by EMS teams was reduced, while the number and percentage of patients seeking help for mental and behavioral health-related complaints increased. At the same time, average times from call to arrival of the medical team as well as from dispatch to arrival at the healthcare facility increased significantly. The current study’s findings support the idea of more effective healthcare, particularly in the emergency healthcare system, with the goal of providing high-quality pre-hospital care and introducing preventive strategies among community mental health services to individuals seeking help for mental or behavioral complaints. Possible interventions aimed at enhancing pre-hospital care will not only benefit the population until the resolution of the ongoing pandemic but will also constitute the cornerstone of future national healthcare systems.

## Data availability statement

The raw data supporting the conclusions of this article will be made available by the authors, without undue reservation.

## Author contributions

NB and JL: conceptualization. NB and SB: methodology. NB: software. NK: validation and formal analysis, data curation, and writing—original draft preparation. NB and AO: writing—review and editing. LS: visualization. NK and LS: supervision. All authors contributed to the article and approved the submitted version.

## Conflict of interest

The authors declare that the research was conducted in the absence of any commercial or financial relationships that could be construed as a potential conflict of interest.

## Publisher’s note

All claims expressed in this article are solely those of the authors and do not necessarily represent those of their affiliated organizations, or those of the publisher, the editors and the reviewers. Any product that may be evaluated in this article, or claim that may be made by its manufacturer, is not guaranteed or endorsed by the publisher.
